# Validating accelerometry estimates of energy expenditure across behaviours using heart rate data in a free-living seabird

**DOI:** 10.1242/jeb.152710

**Published:** 2017-05-15

**Authors:** Olivia Hicks, Sarah Burthe, Francis Daunt, Adam Butler, Charles Bishop, Jonathan A. Green

**Affiliations:** 1 Department of Earth, Ocean and Ecological Sciences, School of Environmental Sciences, University of Liverpool, Liverpool L69 3GP, UK; 2 Centre for Ecology & Hydrology, Bush Estate, Penicuik, Midlothian EH26 0QB, UK; 3 Biomathematics and Statistics Scotland, James Clerk Maxwell Building, The King's Buildings, Peter Guthrie Tait Road, Edinburgh EH9 3FD, UK; 4 School of Biological Sciences, Bangor University, Gwynedd LL57 2UW, UK

**Keywords:** Dynamic body acceleration, Field metabolic rate, Diving, Flying, Shag, *Phalacrocorax aristotelis*

## Abstract

Two main techniques have dominated the field of ecological energetics: the heart rate and doubly labelled water methods. Although well established, they are not without their weaknesses, namely expense, intrusiveness and lack of temporal resolution. A new technique has been developed using accelerometers; it uses the overall dynamic body acceleration (ODBA) of an animal as a calibrated proxy for energy expenditure. This method provides high-resolution data without the need for surgery. Significant relationships exist between the rate of oxygen consumption (*V̇*_O_2__) and ODBA in controlled conditions across a number of taxa; however, it is not known whether ODBA represents a robust proxy for energy expenditure consistently in all natural behaviours and there have been specific questions over its validity during diving, in diving endotherms. Here, we simultaneously deployed accelerometers and heart rate loggers in a wild population of European shags (*Phalacrocorax aristotelis*). Existing calibration relationships were then used to make behaviour-specific estimates of energy expenditure for each of these two techniques. Compared with heart rate-derived estimates, the ODBA method predicts energy expenditure well during flight and diving behaviour, but overestimates the cost of resting behaviour. We then combined these two datasets to generate a new calibration relationship between ODBA and *V̇*_O_2__ that accounts for this by being informed by heart rate-derived estimates. Across behaviours we found a good relationship between ODBA and *V̇*_O_2__. Within individual behaviours, we found useable relationships between ODBA and *V̇*_O_2__ for flight and resting, and a poor relationship during diving. The error associated with these new calibration relationships mostly originates from the previous heart rate calibration rather than the error associated with the ODBA method. The equations provide tools for understanding how energy constrains ecology across the complex behaviour of free-living diving birds.

## Abstract (precis)

**Summary:** A calibration of the overall dynamic body acceleration (ODBA) method for estimating behaviour-specific energy expenditure in terms of *V̇*_O_2__ in free-ranging birds at high temporal resolution.

## INTRODUCTION

Energy is a central currency in the behaviour and physiology of animals (Butler et al., 2004). Individuals have a finite amount of energy to allocate to maximising fitness and hence life history is constrained by energetics (Brown et al., 2004). Such constraints can result in trade-offs between survival and reproduction (Brown et al., 2004; Halsey et al., 2009). By understanding energetics, we are able to gain a more mechanistic understanding of these trade-offs. To achieve this, we need to quantify how energy is allocated and partitioned to different behaviours and processes to understand how life-history decisions are made (Green et al., 2009; Tomlinson et al., 2014), and improve the predictive power of species distribution or population dynamic models (Buckley et al., 2010).

The two main techniques for measuring energy expenditure in the wild are the doubly labelled water method and the heart rate method (Butler et al., 2004; Green, 2011). The doubly labelled water method provides a single estimate of the rate of oxygen consumption (*V̇*_O_2__) over the course of the experiment with no frequency or intensity information (Butler et al., 2004; Halsey et al., 2008). The doubly labelled water technique is a well-accepted method owing to extensive validations and is widely used because of the relative ease of implementation (Butler et al., 2004; Halsey et al., 2008). The heart rate method relies on the physiological relationship between heart rate (*f*_H_) and *V̇*_O_2__, and can provide high-resolution estimates of energy expenditure in free-living animals. However, the *f*_H_ method must be calibrated in controlled conditions and it often involves invasive surgery, particularly for aquatic animals, which can be costly to the animal (Butler et al., 2004; Green, 2011; Green et al., 2009). Information on the behavioural mode of the individual is not inherent or easily estimated in either the doubly labelled water or heart rate methods. Therefore, without extra assumptions (e.g. Portugal et al., 2012; Green et al., 2009) or secondary loggers, they have limited capacity to estimate behaviour-specific energy expenditure.

Recently, a new technique has been developed using accelerometers to measure the overall dynamic body acceleration (ODBA) of an animal as a proxy for energy expenditure (Halsey et al., 2011a; Wilson et al., 2006). Energy costs of animal movement often constitute the majority of energy expended (Karasov, 1992); therefore, body acceleration should correlate with energy expenditure and provide an index of *V̇*_O_2__ (Elliott et al., 2013; Gleiss et al., 2011; Halsey et al., 2011a; Wilson et al., 2006). Significant calibration relationships exist between *V̇*_O_2__ and ODBA across a number of taxa in controlled conditions (Halsey et al., 2008, 2009). Additionally, accelerometer data can provide high-resolution behavioural information (Yoda et al., 2001), presenting an opportunity to estimate the energetic cost of different behaviours in free-living individuals (Halsey et al., 2011a; Wilson et al., 2006). Because of the miniaturisation of accelerometer loggers and their ability to collect high-resolution data without surgery, the use of this technique in the field of ecological energetics has grown substantially in recent years, with research focusing particularly on marine vertebrates (Halsey et al., 2009; Tomlinson et al., 2014; Wilson et al., 2006). However, muscle efficiency may vary across locomotory modes, meaning the relationship between oxygen consumption and accelerometry may also differ among modes (Gómez Laich et al., 2011). In particular, there have been concerns over the use of ODBA as a proxy for energy expenditure during diving, given equivocal results across several air-breathing species in captive and semi-captive conditions (Fahlman et al., 2008a,b; Halsey et al., 2011b). This may be particularly problematic in volant birds as they operate in both air and water, and the higher density and hence resistance of water versus air can dampen movements at the same level of power output (Gleiss et al., 2011; Halsey et al., 2011b). The indirect metabolic costs of hypothermia may also complicate the relationship (Enstipp et al., 2006). These findings contrast with studies that have established the effectiveness of *f*_H_ as a proxy for energy expenditure under similar conditions (Green et al., 2005; White et al., 2011).

As with the heart rate method, calibration of ODBA is required before it can be used to estimate energy expenditure. However, calibrations performed in controlled environments such as treadmills or dive tanks may cause problems for extrapolation to free-living animals, as they do not fully cover the scope of complex natural behaviours (Elliott et al., 2013; Gómez Laich et al., 2011; Green et al., 2009). Given the importance of quantifying the energetic cost of behaviours to understand the fitness consequences in wild populations, it is crucial to validate the accelerometry technique across the natural range of locomotory modes in free-living animals. Validations exist using the doubly labelled water method, which show that ODBA predicts daily averages of energy expenditure (Elliott et al., 2013; Jeanniard-du-Dot et al., 2016; Stothart et al., 2016). However, as the accelerometry technique has developed and is now able to discern and estimate energy expenditure across fine scale behaviours, it is timely to validate these measurements with a technique with equally high resolution (Green et al., 2009).

In this study, we aimed to validate the accelerometry technique against the more established heart rate method in wild free-living European shags, *Phalacrocorax aristotelis* (Linnaeus 1761), a diving seabird species. As calibration relationships exist between *V̇*_O_2__ and ODBA and *f*_H_ for this genus (White et al., 2011; Wilson et al., 2006), we were able to directly compare these estimates of energy expenditure in a free-ranging bird for the first time (Weimerskirch et al., 2016). We simultaneously measured *f*_H_ and acceleration across known behavioural states, including resting, flight and diving, at high temporal resolution, across the natural behavioural range of this diving bird. This allowed us to address the following questions. (1) When using calibration relationships developed in the laboratory, how do estimates of *V̇*_O_2__ derived from ODBA compare with those derived from *f*_H_ at fine temporal scales across behaviours? (2) Is there value in combining what we know from *f*_H_-derived estimates of *V̇*_O_2__ to generate calibration relationships to predict behaviour-specific estimates of ODBA-derived *V̇*_O_2__?

## MATERIALS AND METHODS

The study was carried out on the Isle of May National Nature Reserve, south-east Scotland (56°11′N, 2°33′W) during the breeding season of 2011. European shags are medium-sized foot-propelled diving seabirds that feed benthically on small fish such as sandeel (*Ammodytes marinus*) and butterfish (*Pholis gunnellus*) (Watanuki et al., 2005, 2008)*.* During chick rearing, they typically make 1–4 foraging trips a day (Sato et al., 2008; Wanless et al., 1998). Twelve adult female European shags were captured on the nest during incubation using a crook on the end of a long pole. Females were used to reduce inter-individual variation in *V̇*_O_2__ estimates. Birds were anaesthetised by a trained veterinary anaesthetist (using isoflurane-inhaled anaesthesia) to allow for the implantation of combined acceleration and heart-rate logger devices. This procedure took approximately 60 min and, once recovered, birds were kept for approximately 40 min before being released. Continuous observation of four birds in the field suggested birds resumed normal behaviour in 24 h. Eleven of the 12 instrumented birds were recaptured in the same manner, approximately 35 days later, and anaesthetised to remove the logger. The 12th individual evaded capture as a result of a failed breeding attempt and was recaptured and its logger removed in the 2012 breeding season. Ten birds fledged at least one chick (one brood failed in a storm) in 2011 and the 12th bird successfully bred in 2012. A binomial generalised linear model (GLM) was conducted to compare the breeding success of instrumented birds (*n*=12) with that of uninstrumented birds (*n*=195). Instruments had no significant effect on breeding success (*Z*=0.77, *P*=0.44, d.f.=205). Eight of the 12 loggers were fully functional and recorded from 4 to 33 days of data, totalling 162 days of activity during the breeding season. All studies were carried out with the permission of Scottish Natural Heritage, following the Animals (Scientific Procedures) Act 1986 under UK Home Office licence regulation (PPL 40/3313).

### Instruments

Loggers were custom-built and measured *f*_H_, tri-axial acceleration and depth. The data loggers (50 mm with a diameter of 13 mm, 25 g; 1.6% of the body mass of the sampled individuals, mean±s.d. mass=1561±38 g) and were programmed to store acceleration at 50 Hz, *f*_H_ every second and depth with a resolution of 0.02 m. Devices were sterilised by immersion in chlorhexidine gluconate in alcohol and rinsed in saline.

### Data preparation

Coarse-scale behaviours were categorised from accelerometer data to differentiate between diving, flying and resting (the three main activities of shags) in two steps. First, the ethographer software package (Sakamoto et al., 2009) from IGOR Pro (2000, version 6.3.5; Wavemetrics Inc., Portland, OR, USA) was used to assign data as diving or non-diving behaviour through supervised cluster analysis using *k* means methods on the depth trace (Sakamoto et al., 2009). Second, the remaining accelerometer data was assigned as either flight or resting behaviour (either at sea or on land) using frequency histograms of accelerometer metrics to discriminate between these two coarse-scale behavioural states (Collins et al., 2015). Histograms of standard deviation of the heave axis and pitch (the angle of the device and therefore also of the bird in the surge axis) calculated over 60 s were used to discriminate between flight and rest behaviour:
(1)
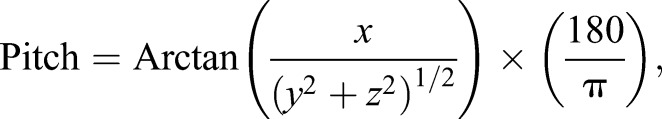
where *x* is acceleration (***g***) in the surge axis, *y* is acceleration (***g***) in the sway axis and *z* is acceleration (***g***) in the heave axis.

ODBA was calculated by first smoothing each of the three acceleration channels with a running mean to represent acceleration primarily due to gravity. In our study, the running mean was 1 s (i.e. 50 data points) as in Collins et al. (2015). The smoothed value was then subtracted from the corresponding unsmoothed data for that time interval to produce a value for ***g*** resulting primarily from dynamic acceleration (Wilson et al., 2006). Derived values were then converted into absolute positive units, and the values from all three axes were summed to give an overall value for dynamic acceleration experienced. Estimates of the rate of oxygen consumption (*V̇*_O_2__, ml min^−1^), were derived from values of both *f*_H_ and ODBA using calibrations conducted in the laboratory on a congeneric species of seabird, the great cormorant, *Phalacrocorax carbo* (see the Appendix for calibration equations; White et al., 2011; Wilson et al., 2006). Great cormorants and European shags are very similar in their geographical ranges, behaviour and physiology; thus, we feel confident that the original calibrations can be used for the European shag. All estimates were ‘whole animal’ as both calibration procedures took intra-individual variation in body mass into account. Locomotory modes included resting, walking and diving during *f*_H_ calibrations, and walking and resting during ODBA calibration. There are no empirical measurements of *V̇*_O_2__ for flight in great cormorants. However, previous estimates of *V̇*_O_2__ during flight from *f*_H_ are comparable to modelled estimates, suggesting that this *f*_H_–*V̇*_O_2__ relationship is robust for flight.

Finally, a dataset was created containing values of ODBA, *f*_H_ and both estimates of *V̇*_O_2__ averaged across each behavioural period per individual, defined as a period of any length of one of the three behavioural states before the next behavioural state begins. We did not constrain the duration of behavioural periods, but took the duration of each period into account during analyses. This dataset was cropped to three full 24 h days during incubation for each individual to keep the duration of data consistent across individuals.

### Data analysis

There were two objectives in the analysis: firstly, to compare ODBA-derived estimates of *V̇*_O_2__ with *f*_H_-derived estimates of *V̇*_O_2__ to investigate whether a one-to-one relationship exists between these two methods (question 1); and secondly, to establish whether a relationship between ODBA- and *f*_H_-derived *V̇*_O_2__ would allow improved prediction of behaviour-specific estimates of *V̇*_O_2__ from accelerometry data at a fine temporal resolution (question 2).

To address question 1 (‘how do ODBA- and *f*_H_-derived estimates of *V̇*_O_2__ compare?’), we modelled *f*_H_-derived *V̇*_O_2__ using linear mixed effects models (LMMs) using the *lme4* package in R (Bates et al., 2014; https://www.r-project.org/). ODBA-derived *V̇*_O_2__ and behavioural state were explanatory variables and we controlled for variation between birds by including individual as a random factor. We fitted models containing all possible combinations of the fixed effects, including models with and without interaction terms (see Table 1). Within each model, observations were weighted by the duration of each behavioural bout divided by the sum of the duration of behavioural bouts for each individual for that behaviour to provide higher weighting to behavioural bouts that are carried out for a longer duration, which represent more generalised behaviours. This ensured that short-lived and/or infrequently expressed behaviours were not over-represented.

**Table 1.**
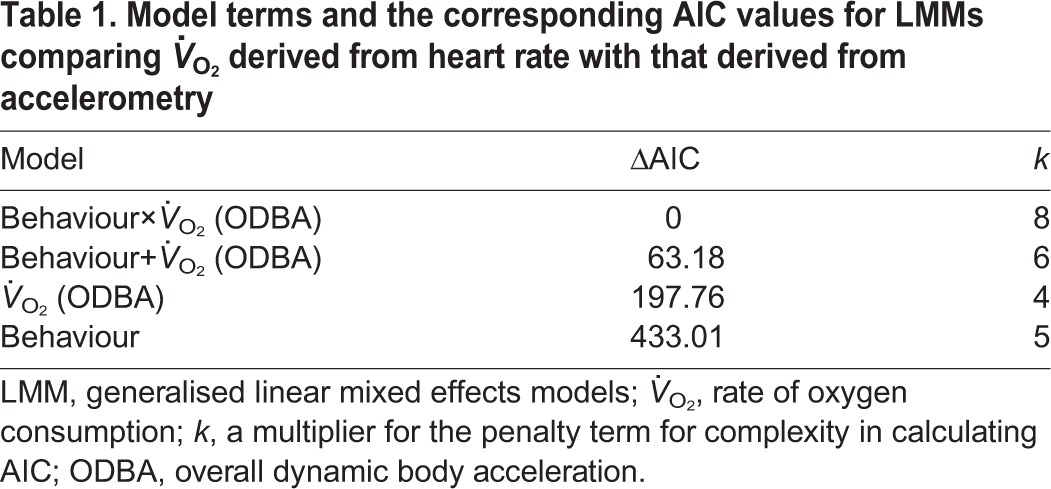
**Model terms and the corresponding AIC values for LMMs comparing *V̇*_O_2__ derived from heart rate with that derived from accelerometry**

To address question 2 (‘is there value in generating calibration relationships between ODBA- and *f*_H_-derived *V̇*_O_2__?’), we created a second set of LMMs. The model structure was the same as before, except that, in the fixed effects part of the model, ODBA-derived *V̇*_O_2__ was replaced with ODBA itself.

In both model sets, model selection was based on Akaike's information criterion (AIC), which penalises the inclusion of unnecessary parameters in models (Burnham and Anderson, 2001). The model with the lowest AIC is usually chosen to be the ‘best’ model, but models within two ΔAIC of the lowest value are generally considered to have similar empirical support to that of the best model. *R*^2^ values were calculated using the MuMIn package in R.

Both ODBA and *f*_H_ are often used to make qualitative comparisons of energy expenditure between, for example, behavioural states or individuals (e.g. Angel et al., 2015; Green et al., 2009). As we aimed to be able to make quantitative estimates and comparisons of *V̇*_O_2__ using ODBA (question 2), we needed to incorporate the error associated with the conversion from *f*_H_ to *V̇*_O_2__ into our predictions. To quantify this, we developed a bootstrapping approach, which we implemented separately for each behavioural state. For each state, we used a fitted model of *f*_H_ as a function of ODBA to simulate 100 possible *f*_H_ values for given values of OBDA: these *f*_H_ values were drawn from a normal distribution with mean equal to the estimated value of *f*_H_ (based on the fitted model) and standard deviation equal to the standard error of the estimate (SEE) that was produced by the fitted model. For each of these *f*_H_ values, we then simulated 100 values of *V̇*_O_2__ using the fitted equation, and associated SEE, from White et al. (2011). This gives a total of 10,000 simulated values of *V̇*_O_2__ for each value of ODBA. We took the mean of these values to be our estimate for the value of *V̇*_O_2__, for each value of OBDA, and used the 2.5% and 97.5% quantiles to give us the associated 95% confidence limits. Both sets of SEE calculations assumed 100 measurements of ODBA from each of 10 individuals; these were assumed to be a typical sample size of individuals and average number of ODBA measurements per individual. These error distributions are calculated to enable the calibrations to be used with quantifiable error associated with the predictions. See Green et al. (2001) for a full description of how SEE calculations are made.

## RESULTS

### Comparison of oxygen consumption estimates

**Fig. 1.**
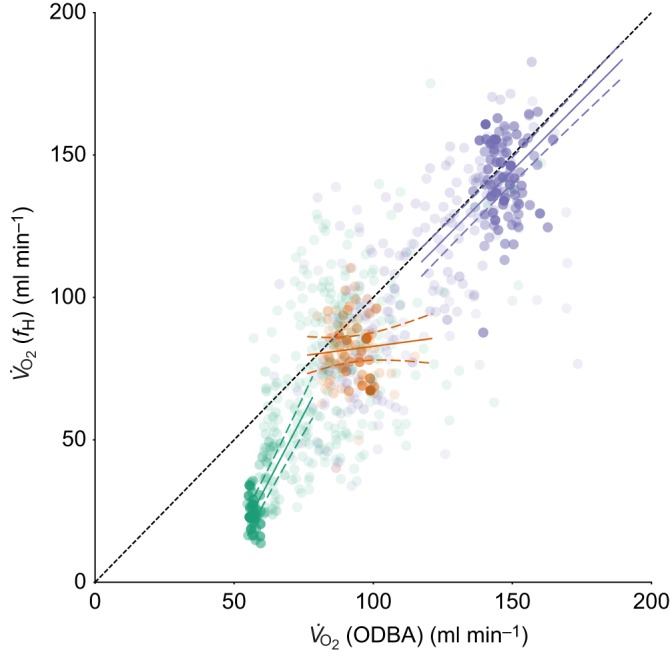
**The relationship between the two methods for predicting the rate of oxygen consumption (*V̇*_O_2__) across different behavioural states.** The dotted line represents equality between the heart rate (*f*_H_) and overall dynamic body acceleration (ODBA) methods. Behaviour-specific regression relationships (solid line) and 95% confidence intervals (dashed lines) for each behaviour (resting in green, diving in orange and flying in purple) are shown. Points vary in transparency according to the duration of time represented by each behavioural bout. The horizontal and vertical range of the regression lines indicates data points encompassing 99% of the entire duration of time spent in each behaviour.

There was a positive relationship between *f*_H_-derived *V̇*_O_2__ and ODBA-derived *V̇*_O_2__ (Fig. 1). The best model included an interaction between ODBA-derived *V̇*_O_2__ and behaviour (Table 1), suggesting a difference among behaviours in the relationship between oxygen consumption estimates. Pairwise comparisons revealed differences among all three behaviours in the relationships between the estimates of *V̇*_O_2__ made using the two techniques. The best overall model was a good fit (marginal *R*^2^=0.70); however, *R*^2^ values for behaviour-specific relationships were much lower (Table 2). When the behaviours were considered individually, there was a positive relationship for flying and resting and but no relationship for diving (Table 2). Estimates of *V̇*_O_2__ from both *f*_H_ and ODBA showed considerable variability but sat close to the line of equality for flight and diving behaviour. However, ODBA-based estimates of *V̇*_O_2__ were consistently greater than those estimated by *f*_H_ (Fig. 1). There was relatively little variability in ODBA-derived *V̇*_O_2__ during resting behaviour; this can be attributed to similarly little variability in raw ODBA values (Fig. S1).

**Table 2.**
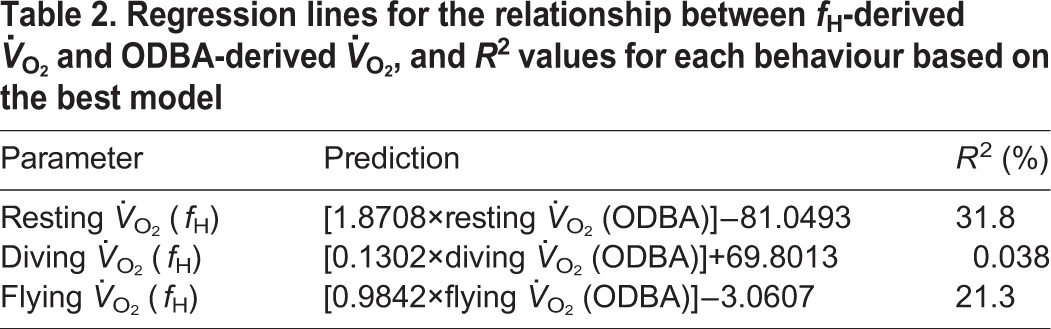
**Regression lines for the relationship between *f*_H_-derived *V̇*_O_2__ and ODBA-derived *V̇*_O_2__, and *R*^2^ values for each behaviour based on the best model**

### ODBA as a predictor of *V̇*_O_2__

**Fig. 2.**
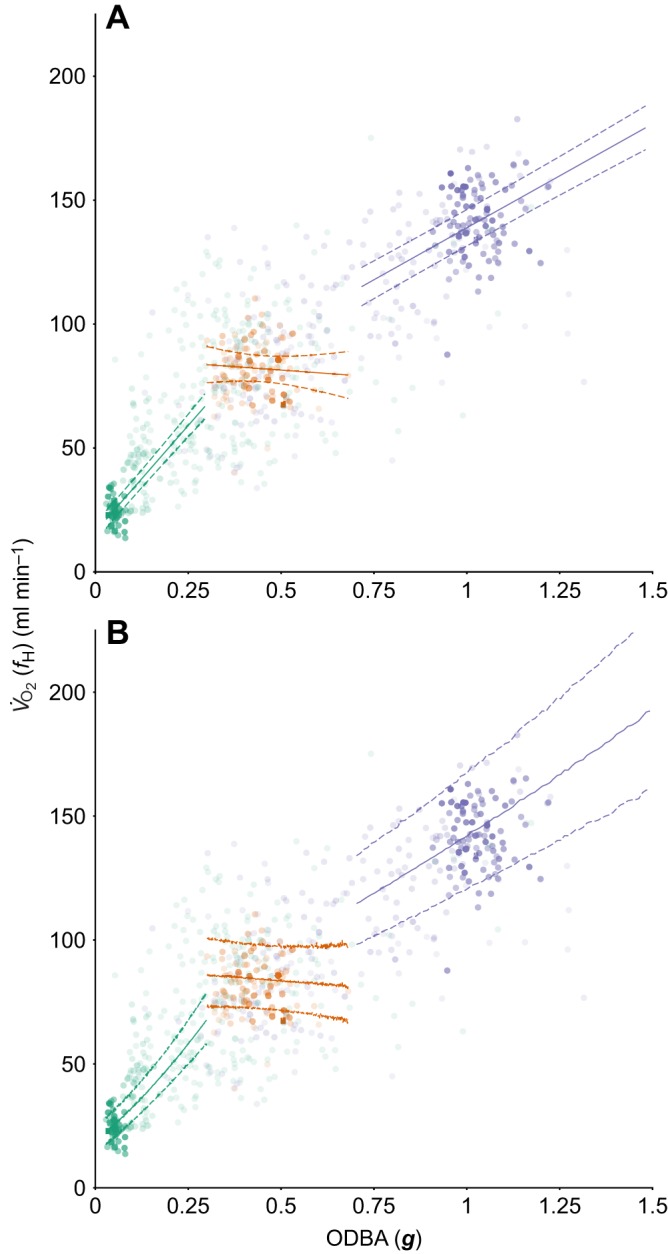
**The relationship between ODBA- and *f*_H_-derived energy expenditure.** Behaviour-specific regression relationships (solid line) and 95% confidence intervals (dashed lines) for each behaviour (resting in green, diving in orange and flying in purple) are shown. Point transparency varies with duration of time spent in each behavioural bout. (A) The 95% confidence intervals are taken from the model estimates without taking into account the residual error associated with converting *f*_H_ to *V̇*_O_2__ estimates. (B) The 95% confidence intervals from the bootstrapping method accounting for the residual error associated with converting *f*_H_ to *V̇*_O_2_ _estimates.

When using ODBA as a predictive tool for estimating energy expenditure, there was a positive relationship between ODBA- and *f*_H_-derived *V̇*_O_2__. The best model fitted an interaction between ODBA and behaviour (Table 3). Examination of behaviour-specific relationships (Fig. 2) suggests that ODBA is a useable proxy of *V̇*_O_2__ during flying and resting, but a poor proxy for diving (see Table 4 for behaviour-specific predictive equations). When accounting for the residual error associated with the *f*_H_ *V̇*_O_2__ calibration, it is evident that a large amount of error is associated with the laboratory calibration between *f*_H_ and *V̇*_O_2__. Indeed, most of the uncertainty in predicting *f*_H_-derived *V̇*_O_2__ from ODBA arises from the uncertainty in the calibration of the *f*_H_ technique rather than from the estimation of the correlation between the two techniques (Fig. 2).

**Table 3.**
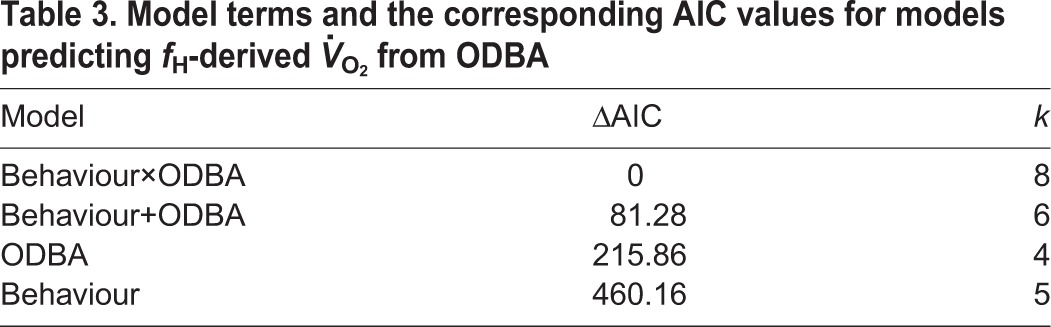
**Model terms and the corresponding AIC values for models predicting *f*_H_-derived *V̇*_O_2__ from ODBA**

**Table 4.**
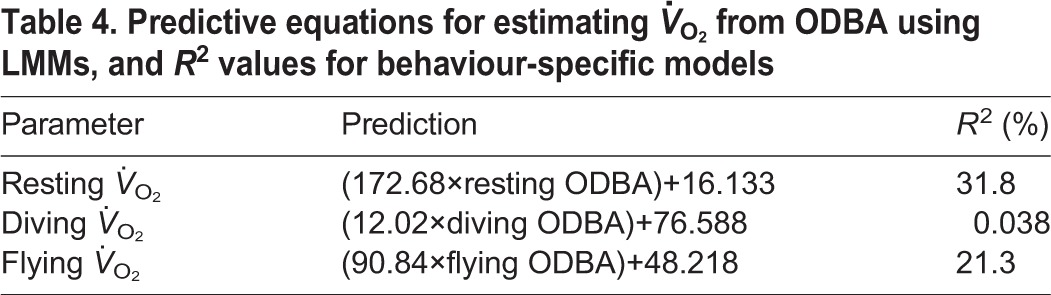
**Predictive equations for estimating *V̇*_O_2__ from ODBA using LMMs, and *R*^2^ values for behaviour-specific models**

## DISCUSSION

Relatively few studies have investigated whether ODBA represents a robust proxy for energy expenditure across natural behaviours at high resolution in free-ranging birds (Duriez et al., 2014; Weimerskirch et al., 2016). Here, we compared energy expenditure estimates across a range of natural behaviours in a free-living organism using both the established *f*_H_ method and accelerometry. Across behaviours, we found a good relationship between ODBA and *V̇*_O_2__. Within individual behaviours, we suggest that ODBA is a useable proxy of energy expenditure during flying and resting, thus opening up potential new avenues of research for quantifying energy budgets for individuals across key behaviours. However, some caution is necessary: we found that ODBA is less reliable at estimating energy expenditure during diving behaviour, though this may be due in part to lower variation in ODBA during diving than within flight or resting. We combined these findings to provide usable behaviour-specific calibration relationships between ODBA and *V̇*_O_2__ to more accurately estimate energy expenditure using the accelerometry technique alone.

### Comparison of oxygen consumption estimates

Whilst there was a good relationship between the estimates made with the two approaches, and ODBA estimates of *V̇*_O_2__ for flight and diving sit well on the line of equality, ODBA overestimates *f*_H_-derived *V̇*_O_2__ for resting behaviour. It is known that ODBA estimates of energy expenditure during inactivity tend to be poorer than those in high activity because movement makes up a small proportion of energy expenditure during inactivity (Green et al., 2009; Weimerskirch et al., 2016). Differences in estimates across the two techniques for resting may also have arisen because the underlying laboratory-based calibrations with *V̇*_O_2__ that underpin our estimates were undertaken in different conditions. Although both ODBA-derived *V̇*_O_2__ and *f*_H_-derived *V̇*_O_2__ lab calibrations for great cormorants (*P. carbo*) were based on the same captive individuals, they were conducted in different seasons (November and March/June, respectively) (Gómez Laich et al., 2011; White et al., 2011; Wilson et al., 2006). Seasonal variation in basal metabolic rate (BMR) is well documented (Smit and McKechnie, 2010). In this case, the cormorants had lower BMR in the summer months (C. R. White, P. J. Butler and G. P. Martin, unpublished data). The higher resting *V̇*_O_2__ values estimated by ODBA compared with *f*_H_ may be due to the higher resting metabolic rate incorporated into the ODBA calibration. Thus, as ODBA is not sensitive to changes in BMR and cannot record seasonal variation in metabolic rate, this may be a limitation to this approach in studies trying to estimate seasonal changes in energy expenditure within a population or species. A strength of the approach described here is that because the *f*_H_/*V̇*_O_2__ calibrations were made during the summer, our new predictive equations allow *V̇*_O_2__ to be estimated from ODBA during the summer months, thus accounting for seasonal changes in BMR.

Estimates of flight costs were lower than expected based on body mass alone (Bishop et al., 2002) but consistent with previous estimates based on calibrations from a congeneric species, the great cormorant (White et al., 2011). It is possible that both *f*_H_ and ODBA underestimate *V̇*_O_2__ during flight as Ward et al. (2002) show that *V̇*_O_2__ during flight in two species of geese would be underestimated based on *f*_H_ during flight and a walking-only calibration relationship. This is due to differences in calibration relationships for walking and flying in these species of geese. However, in great cormorants, the original calibration line between *f*_H_ and *V̇*_O_2__ intersects with modelled estimates of flight *V̇*_O_2__, suggesting the *f*_H_–*V̇*_O_2__ relationship is robust for flight (Bishop et al., 2002; White et al., 2011). Additionally, the close agreement of our ODBA- and *f*_H_-derived estimates of *V̇*_O_2__ during flight suggests that ODBA-based estimates are also accurate. This is either a coincidence or provides support for the previous papers and methodologies. However, more research on the true costs of flight in unrestrained birds under natural conditions is urgently needed (Elliott, 2016).

### ODBA as a predictor of energy expenditure

We found ODBA to be a good predictor of *V̇*_O_2__; our best overall model, which includes the effect of behaviour, is comparable to other studies and calibrations, suggesting there is considerable value in this method when used across a range of behaviours. The *R*^2^ value for our overall model is comparable but slightly lower than that for studies comparing partial dynamic body acceleration and energy expenditure by the doubly labelled water method in the wild [*R*^2^=0.73 in thick billed murres (Elliott et al., 2013) and *R*^2^=0.91 in pelagic cormorants (Stothart et al., 2016)] and consistently lower than measurements obtained on treadmills in the laboratory [*R*^2^=0.81–0.93 for four bird and mammal species (Halsey et al., 2009)] and experimental dive tanks [*R*^2^=0.83 for green turtles (Enstipp et al., 2011)]. The *R*^2^ value from this study is expected to be lower than those from previous studies, as the ODBA values were not daily averages as in most previous studies, but instead were calculated over shorter time scales of behavioural bouts (Elliott, 2016; Green, 2011). However, when our data were re-examined over a daily scale, the *R*^2^ value for the best overall model (marginal *R*^2^=0.97) was higher than our calibration at finer temporal scale and more similar to previous calibrations using daily averages (see Figs. S2, S3 and Table S1).

### Behavioural differences

The high temporal resolution of the calibration in this study compared with that of previous studies (Elliott et al., 2013; Jeanniard-du-Dot et al., 2016; Stothart et al., 2016) allows the more complex differences in energy expenditure between behaviours and resultant differences in predictive estimation equations between different behaviours to be quantified. All three behavioural modes had different predictive equations when estimating *V̇*_O_2__ from ODBA. Similarly, in calibrations of daily energy expenditure using the doubly labelled water method in the field, Elliott et al. (2013) and Stothart et al. (2016) found the most parsimonious models included classification of one behaviour separately from the others. The difference in our study, however, is that the best model includes all behaviours separately. This may be driven by how well ODBA is able to reflect metabolic costs of movement in different media. ODBA provided reasonable estimates of *V̇*_O_2__ in flight, which is not unexpected given ODBA has been shown to correlate with *f*_H_ in previous studies [frigate birds (Weimerskirch et al., 2016) and griffons (Duriez et al., 2014)]. This is further supported by correlates between wing beat frequency and *f*_H_ in bar headed geese (Bishop et al., 2015), which have a similar flapping flight to European shags. There is also evidence from studies that one calibration of energy expenditure can be applied to all behavioural modes, though these studies did not involve diving or flying behaviour (Green et al., 2009; Wilson et al., 2006).

ODBA provided poorer estimates of *V̇*_O_2__ during diving, which supports the finding of Halsey et al. (2011b) that ODBA did not correlate with oxygen consumption over diving bouts in double crested cormorants in dive tank experiments (Halsey et al., 2011b). Cormorant species have partially wettable plumage (Grémillet et al., 2005), which causes high rates of heat loss and therefore high dive costs (Enstipp et al., 2005). As a result, they may be susceptible to changes in metabolic rate within diving bouts (Enstipp et al., 2006; Gremillet et al., 1998), which would be expressed as changes in *f*_H_ but not in ODBA, producing no clear relationship between ODBA and *V̇*_O_2__.

### Application of findings

By incorporating the error associated with the *f*_H_-derived *V̇*_O_2__ calibration (White et al., 2011), we were able to derive relationships for each behaviour to predict oxygen consumption and its associated error from ODBA values. It is notable that it is the error originating from the laboratory-based calibration between *f*_H_ and *V̇*_O_2__ that is driving the large error distribution overall, rather than the comparison between *f*_H_ and ODBA in the field. As the ODBA technique for measuring energy expenditure is becoming increasingly popular in the field, and provides fine-scale information on the behaviour of the animal, it is essential to be able to use behaviour-specific equations as this currently accounts for most of the uncertainty in free-living animal energy budgets (Collins et al., 2016; Wilson et al., 2006). Our validation exercise indicates that for an average day, our approach gives broadly similar estimates of energy expenditure to those derived from first principles and the literature (Fig. S4). The behavioural-bout resolution of our calibration provides a natural range of behavioural bouts of varying lengths, created with free-ranging birds and natural behavioural bouts, meaning this calibration can be used at any temporal scale for resting and flight behaviour. While it is not possible to present a single equation that captures both elements of the residual error associated with predictions, we provide a script that calculates estimates with SEE for a given value of ODBA (please contact the corresponding author).

This study therefore outlines an approach to generate behaviour-specific estimates of energy expenditure from ODBA, which can be used to more accurately estimate total energy expenditure in the complex behaviour of free-living cormorant species. However, the poor predictive power of ODBA during diving reinforces the proposal that further temporal considerations may need to be incorporated for this behaviour. Whilst future recommendations include the simultaneous measurement of *f*_H_, acceleration and *V̇*_O_2__ with respirometry, we have provided equations that combine both *f*_H_ and ODBA techniques as predictors of behaviour-specific energy expenditure. ODBA-derived behaviour-specific estimates of energy expenditure can help pave the way for future work answering ecologically important questions and understanding the fine-scale costs of movement and foraging of diving seabirds.

## APPENDIX

### Existing calibration equations used in analyses

Calibration equation from Wilson et al. (2006) for the relationship between ODBA and *V̇*_O_2__:

(A 1)

Calibration equation from White et al. (2011) for the relationship between *f*_H_ and *V̇*_O_2__:

(A 2)

where *M* is mass.
